# Comparative Outcomes of Transoral Robotic Surgery Versus Open Surgery in Head and Neck Cancer: A Systematic Review and Meta-Analysis

**DOI:** 10.7759/cureus.116164

**Published:** 2026-09-13

**Authors:** Anfal B Basheer, Ghada A Al-Abdullah, Eman A Alzaher, Rahaf H Alharbi, Mohammed S Aljabri, Ghadah Khalid H Alanazi, Mohammed A Alharbi, Ghaida Y Alsahli, Yousef H AlYousef, Maha Al-Gilani

**Affiliations:** 1 Otolaryngology - Head and Neck Surgery, Sheikh Jaber Al-Ahmad Hospital, Kuwait City, KWT; 2 College of Medicine, King Saud bin Abdulaziz University for Health Sciences, Riyadh, SAU; 3 College of Medicine, King Abdulaziz University, Jeddah, SAU; 4 General Practice, Jeddah Second Health Cluster, Jeddah, SAU; 5 General Practice, Northern Borders Health Cluster, Arar, SAU; 6 College of Medicine, Imam Abdulrahman Bin Faisal University, Dammam, SAU; 7 University of Medicine and Health Sciences, Royal College of Surgeons in Ireland, Dublin, IRL

**Keywords:** head and neck neoplasms, meta-analysis, minimally invasive surgery, oncologic outcomes, open surgery, oropharyngeal cancer, oropharyngeal squamous cell carcinoma, systematic review, transoral robotic surgery

## Abstract

Oropharyngeal squamous cell carcinoma (OPSCC) represents a growing global health concern, particularly with the rising incidence of human papillomavirus-associated disease. Transoral robotic surgery (TORS) has emerged as a minimally invasive alternative to conventional open approaches, offering enhanced visualization and potentially reduced morbidity compared with mandibulotomy and transcervical techniques.

This systematic review and meta-analysis aimed to compare functional, oncological, and postoperative outcomes between TORS and open surgery in patients with OPSCC. The review was conducted in accordance with PRISMA 2020 guidelines and registered in PROSPERO (CRD42024616159). A comprehensive search of PubMed, Cochrane Library, Google Scholar, and ClinicalTrials.gov from database inception to November 2024 identified eligible comparative studies. Data extraction and risk-of-bias assessment were performed independently by two reviewers, and meta-analysis was conducted using RevMan 5.3. Seven studies comprising 15,907 patients were included, of whom 2,164 underwent TORS and 13,742 underwent open surgery. TORS was associated with improved functional outcomes, including lower tracheostomy rates, shorter hospital stay, and reduced feeding tube dependence. Recurrence rates and overall survival favored TORS, while postoperative complications and positive margin rates were comparable between groups. Despite moderate heterogeneity and the absence of randomized trials, these findings suggest that TORS provides meaningful functional and oncological advantages. Further high-quality randomized studies are warranted to confirm long-term outcomes and optimize patient selection.

## Introduction and background

Head and neck cancers, particularly oropharyngeal squamous cell carcinomas (OPSCC), constitute a major global health concern due to their rising incidence and profound impact on patients’ quality of life. Human papillomavirus (HPV)-positive OPSCC has emerged as a unique clinical entity distinct from its HPV-negative counterpart. Unlike HPV-negative tumors, which are traditionally associated with smoking and alcohol use, HPV-positive OPSCC is driven by HPV infection, most commonly HPV-16. This form of OPSCC most commonly affects younger, nonsmoking individuals and is linked to a more favorable prognosis due to its higher sensitivity to radiation and chemotherapy [1-3].

Transoral robotic surgery (TORS) has emerged as a minimally invasive approach designed to address both oncologic control and functional preservation in the surgical management of head and neck cancers. The origins of TORS date back to the early 2000s, when Weinstein and O’Malley adapted the da Vinci surgical system for transoral use [3,4]. Its approval by the U.S. Food and Drug Administration (FDA) in 2009 for the treatment of T1 and T2 oropharyngeal SCC marked a milestone in surgical oncology [2,3,5].

Unlike open surgical approaches that often involve invasive procedures, such as mandibulotomy or transcervical incisions, TORS provides transoral access to anatomically complex regions, allowing precise excision with minimal disruption to surrounding structures [3,6,7].

The advantages of TORS are well-documented. Studies consistently showed shorter operative time, reduced hospital stay, lower rates of complications, and faster recovery of critical functions such as swallowing and speech [1,4,6]. Oncologically, TORS achieves comparable or superior rates of margin negativity and local control, with reduced reliance on postoperative chemoradiotherapy [2,5,8].

Moreover, the minimally invasive nature of TORS reduces the physical and psychological burden on patients, contributing to improved postoperative quality of life [6,9]. Despite these benefits, TORS is not without limitations. The technique requires significant investment in robotic equipment and specialized training, presenting barriers to widespread adoption [3,4]. In addition, its utility is constrained in advanced-stage tumors or anatomically complex cases, where open surgical approaches remain the gold standard. Open surgery, though effective, is associated with longer recovery times, higher morbidity, and greater functional impairment [7,9].

While previous studies have compared TORS with open surgery, uncertainty remains regarding their relative oncologic and functional outcomes, with inconsistent findings reported across the available literature. Therefore, this systematic review and meta-analysis aimed to compare the functional and oncological outcomes of TORS and open surgery in patients with OPSCC.

## Review

Methods 

Review of the Literature

This systematic review was prospectively registered in PROSPERO (CRD42024616159) [10]. It was conducted in adherence to the Preferred Reporting Items for Systematic Reviews and Meta-Analyses (PRISMA) guidelines [11]. A comprehensive electronic search was conducted in PubMed, Google Scholar, Cochrane Central Register of Controlled Trials (CENTRAL), and online trial registers, including the ClinicalTrials.gov database for studies published from inception to November 2024. Further articles were found by examining the reference lists of the reviewed studies. A search was conducted using the following search strategy: (Transoral robotic surgery OR TORS) AND (Open surgery OR salvage surgery OR conventional surgery OR Open approach to the oropharynx OR Traditional Surgery) AND (head and neck cancer OR head and neck neoplasm OR oral cancer OR Oropharyngeal Squamous cell carcinoma OR OPSCC OR Squamous cell carcinoma of head and neck). Studies were included in the review based on the PICOSTS criteria, which encompass population, intervention, comparison, outcome, timing, and setting [12].

Methodology for Selecting Studies

Criteria for inclusion in our study were as follows: (1) studies published in English only, (2) study design was one of the following: retrospective or prospective observational studies and randomized and non-randomized controlled trials, (3) patients diagnosed with OPSCC, (4) patients managed with transoral robotic surgery or open surgery, (5) studies that reported some of the functional outcomes (length of hospitalization, decannulation time, and swallowing ability) and the oncological outcomes (recurrence, disease-free years, and overall survival rate), as well as intraoperative and postoperative complications.

Process of Screening and Data Extraction

The outputs of electronic database searches were imported into Rayyan Software for screening, selection, and resolution of any duplicates [13]. Two authors individually assessed the titles and abstracts of all studies identified in the database search to identify potentially eligible studies. Subsequently, a full-text review of the articles was conducted by two reviewers simultaneously. Any disagreements were resolved by a third reviewer. Data extraction was conducted by two reviewers and included the following variables: (1) Demographic information: total number of patients who underwent either the TORS or open surgical approach, along with age, gender, smoking status, and alcohol consumption; (2) Disease-specific variables: HPV status, primary tumor subsite, tumor histology, TNM staging, overall cancer stage, and the number of patients who underwent free-flap reconstruction; (3) Functional outcomes: number of patients who required tracheostomy tube replacement at preoperative, intraoperative, and postoperative stages, decannulation time, hospitalization duration, swallowing ability, the number of patients who needed a feeding tube, and the duration of feeding tube use; (4) Oncological outcomes: duration of follow-up (in months), number of patients with recurrence, disease-free years, and overall survival rate; (5) Intraoperative complications: intraoperative bleeding, amount of blood loss, and operative time; (6) Postoperative complications: postoperative airway edema, postoperative bleeding, postoperative fistula, flap necrosis, cervical hematoma, and swallowing disorders; (7) Postoperative adjuvant therapy: number of patients who received radiotherapy, chemotherapy, or chemoradiotherapy. Two reviewers resolved any disagreement in data extraction.

Assessment of Quality and Bias Risk

All included studies were evaluated for quality and bias based on their design. The quality assessment was performed using the Methodological Index for Non-Randomized Studies (MINORS), a validated tool for assessing the methodological quality of non-randomized studies, including cohort, case-control, and comparative observational studies [14]. The Appraisal Tool for Cross-Sectional Studies (AXIS) was employed for cross-sectional studies. AXIS consists of 20 items, each typically answered as "Yes", "No", or "Unclear" [15]. A higher number of "Yes" responses indicates better methodological quality and a lower risk of bias. Additionally, the studies were independently assessed by two reviewers.

Meta-analysis of the Included Data

The meta-analysis was done using Review Manager (RevMan), version 5.3, developed by The Nordic Cochrane Centre, The Cochrane Collaboration. For continuous outcomes, mean differences (MD) with 95% confidence intervals (CI) were calculated, while for dichotomous outcomes, odds ratios (OR) with 95% CI were used. Heterogeneity was assessed using the I² statistic. For studies exhibiting moderate to high heterogeneity (I² ≥ 50%), a random-effects model was applied to account for variability between studies. Conversely, for studies with low heterogeneity (I² < 50%), a fixed-effects model was employed.

Results

Literature Findings

**Figure 1:**
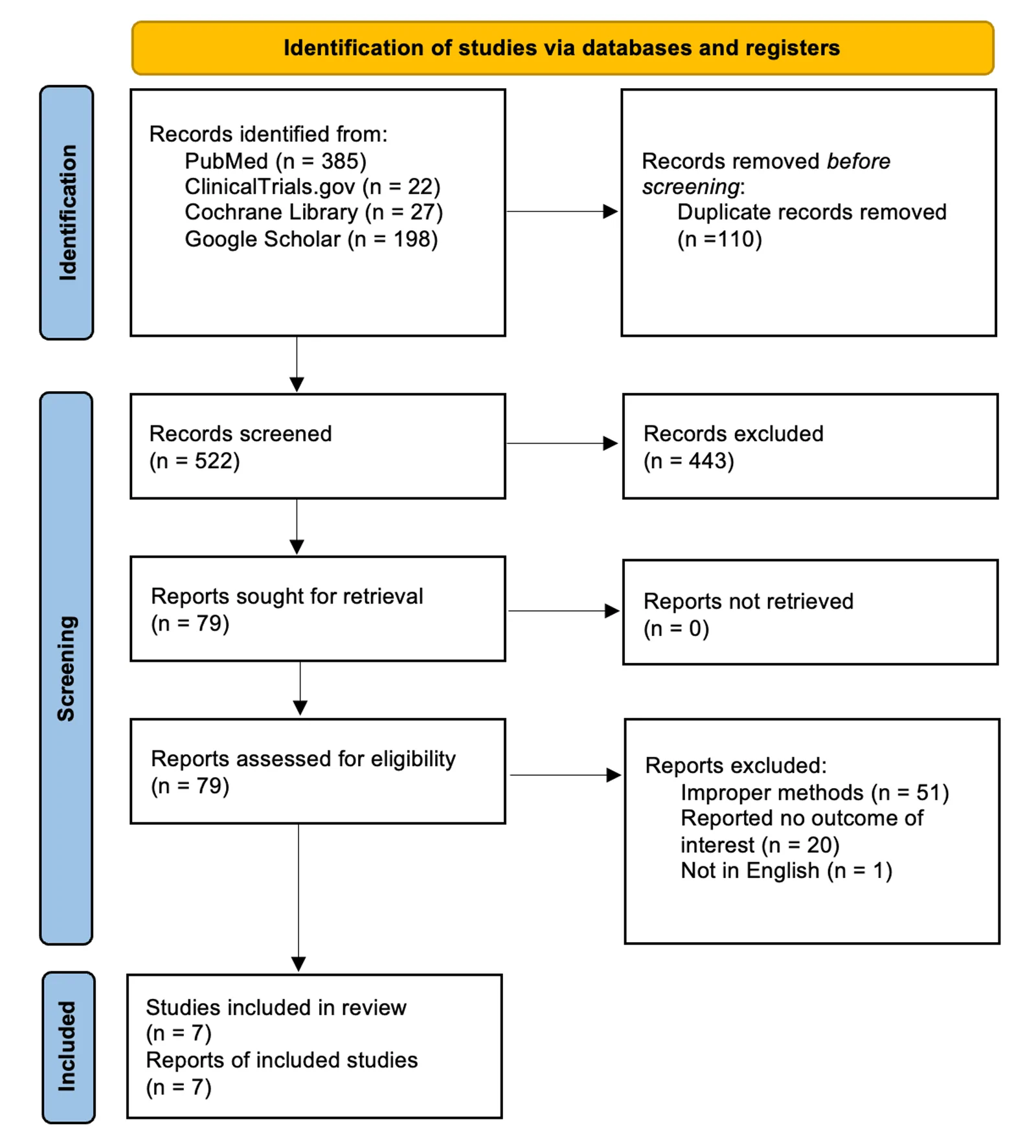
PRISMA flow diagram of literature screening

Our systematic search identified 632 publications, with 385 sourced from PubMed, 198 from Google Scholar, 27 from the Cochrane Library, and 22 from ClinicalTrials.gov. After the removal of duplicate articles, we identified a total of 522 publications. Initially, we retrieved 79 full-text articles for this systematic review. However, after applying the exclusion criteria, only seven articles were included from the beginning until November 2024. The PRISMA flow diagram illustrating the study selection process is presented in Figure 1 [1,2,4-6,9,16].

Among these, there were five cohort studies, one case-control study, and one cross-sectional study. Notably, none of the selected studies were randomized controlled trials. The fundamental characteristics of the included studies are summarized in Table 1.

**Table 1:** Basic features of the included studies Sources: [1,2,4-6,9,16]

Authors	Year	Type of analysis	Country	Sample Size
Porcuna et al. [2]	2023	Retrospective	Spain	30
White et al. [6]	2013	Retrospective	USA	128
Lee et al. [16]	2013	Prospective	South Korea	57
Shenouda et al. [5]	2020	Retrospective	France	45
Motz et al. [1]	2017	Retrospective	USA	3573
Chung et al. [9]	2014	Retrospective	USA	6944
M. Chen et al. [4]	2014	Retrospective	USA	5146

Description of the Characteristics of the Included Studies

In total, 15,907 patients were identified across the studies included in this systematic review and meta-analysis. Of these, 2,164 patients underwent transoral robotic surgery (TORS), while 13,742 opted for an open surgical approach. The participants’ mean age ranged from 58 to 64 years. Among them, 11,344 were male (71%), and 4,572 were female (29%). Additionally, 1,765 patients tested positive for HPV, while 799 were HPV-negative. The studies reported that 4,775 patients had squamous cell carcinoma, while six patients in the Shenouda study and 331 in the M. Chen study were diagnosed with basal squamous carcinoma [4,5]. The distribution of primary tumor subsites varied between the surgical methods and the TORS group. In the open surgical cohort, most tumors were found in the anterior tongue, followed by the tonsils, tongue base, and oropharyngeal wall. In contrast, in the TORS group, the most prevalent primary tumor site was the oropharyngeal wall, followed by the tonsils, tongue base, and anterior tongue. The analysis of cancer stages in the TORS and open surgical groups revealed similar distributions across stages. Moreover, a total of 77 patients who underwent the open surgical approach required free-flap reconstruction, compared to only 15 patients in the TORS group who needed this type of reconstruction. In one study, only the mandibulotomy subgroup was included in the meta-analysis to avoid bias associated with combining different open surgical approaches, particularly due to significant functional and recovery differences between the transoral and mandibulotomy methods [16]. Table 2 summarizes the baseline characteristics of the included studies.

**Table 2:** Baseline characteristics of the included studies NA = Not applicable Sources: [1,2,4-6,9,16]

Authors	Groups	Number of participants	Total	Sex: Female: Male (for both groups)	Age, mean (for both groups)	HPV positivity
Porcuna et al. [2]	TORS	15	30	8: 22	64.4	2
	Open surgical	15				1
White et al. [6]	TORS	64	128	27: 101	61	NA
	Open surgery	64				NA
Lee et al. [16]	TORS	27	57	11: 46	57.8	18
	Mandibulotomy	14				11
	Transoral	16				10
Shenouda et al. [5]	TORS	21	45	17: 28	61.5	7
	Open surgery	24				7
Motz et al. [1]	TORS	304	3573	832: 2741	NA	NA
	Open surgery	3268				NA
Chung et al. [9]	TORS	856	6944	2535: 4409	59.2	26
	Open surgery	6088				101
M. Chen et al. [4]	TORS	877	5146	1145: 4010	59	424
	Open surgery	4269				1158

Quality Assessment of Included Studies

**Figure 2:**
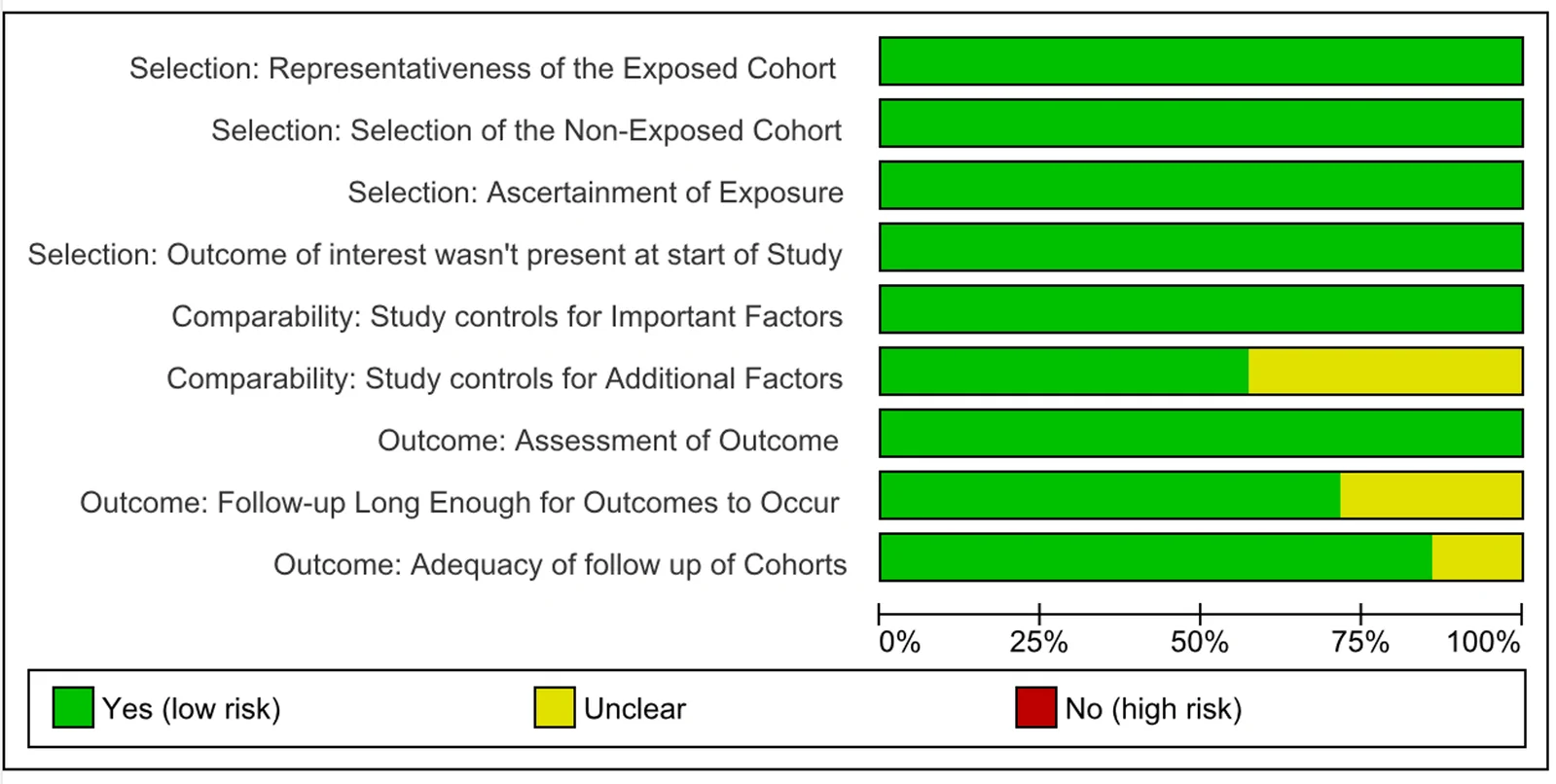
Risk-of-bias graph: methodological index for non-randomized studies assessment tool Review authors' judgments about each risk-of-bias item presented as percentages

The methodological quality of included studies was assessed by two independent reviewers, and any disagreement was resolved by a third senior reviewer. Figure 2 demonstrates that the methodological quality of the included cohort studies was generally high.

**Figure 3:**
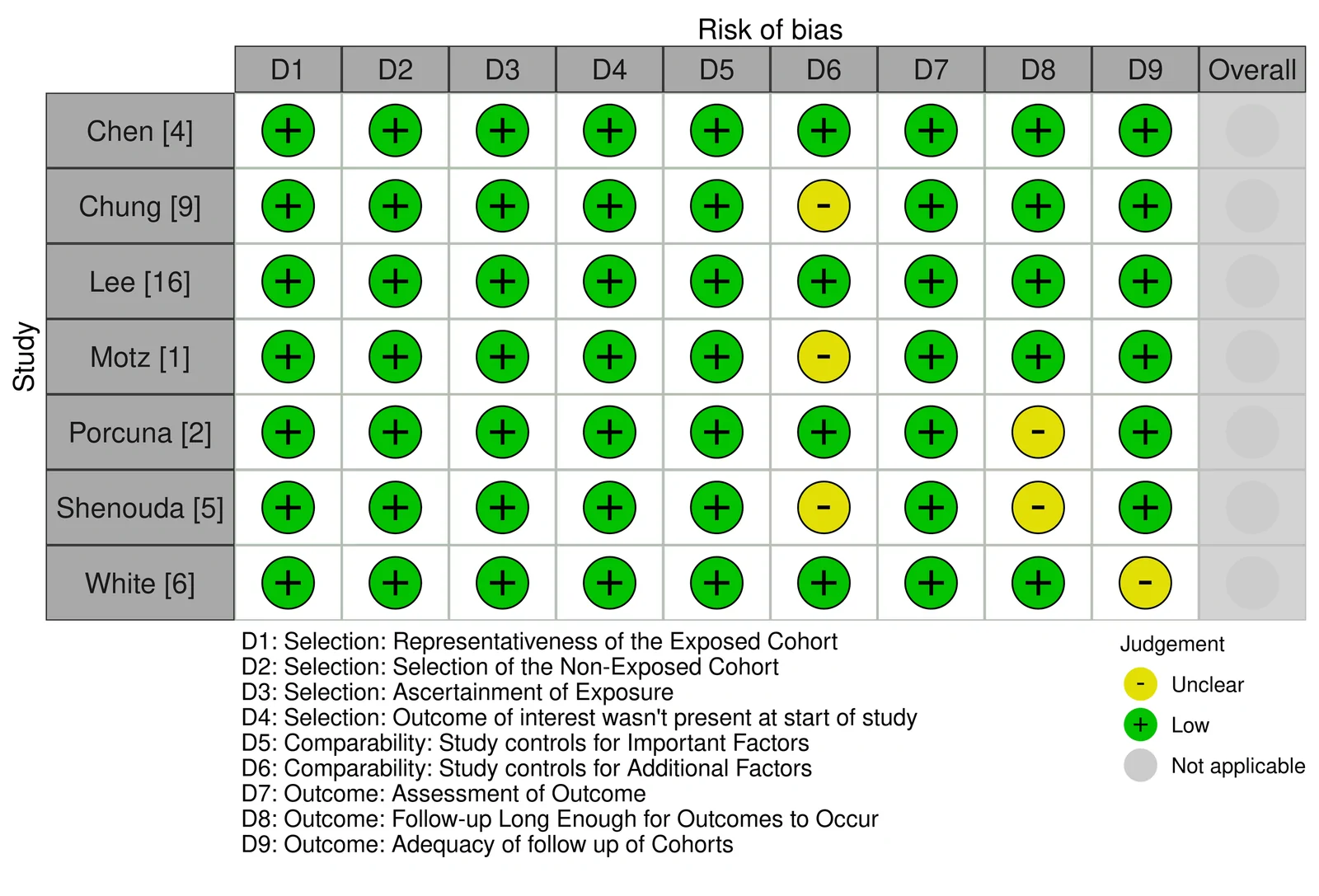
Total methodological index for non-randomized studies (MINORS) score for the included studies plotted against publication year Sources: [1,2,4-6,9,16]

All studies showed lower risk of bias for important domains, such as representativeness of the exposed cohort, appropriate selection of the non-exposed cohort, secure ascertainment of exposure, and confirmation that the outcome of interest was not present at the start of the study, which indicated well-defined and comparable study populations. The control for the important confounding factors was consistently reported, although adjustment for additional confounders was unclear in a proportion of studies, which suggests some residual confounding may remain. The assessment of the outcome was robust across all studies, which showed reliable and objective measurement of endpoints. The duration of follow-up was also adequate in most studies; however, there was a minority that had unclear follow-up length or completeness, which could introduce attrition bias. In terms of detailed quality assessment of the individual studies, Figure 3 shows that three studies achieved a low risk of bias across all assessed domains [4,6,16]. Two studies had unclear adjustment for additional confounders [1,9]. One study showed uncertainty regarding confounder control and follow-up duration [5]. Overall, study quality remained high with only minor methodological concerns.

Assessment of Publication Bias and Heterogeneity

**Figure 4:**
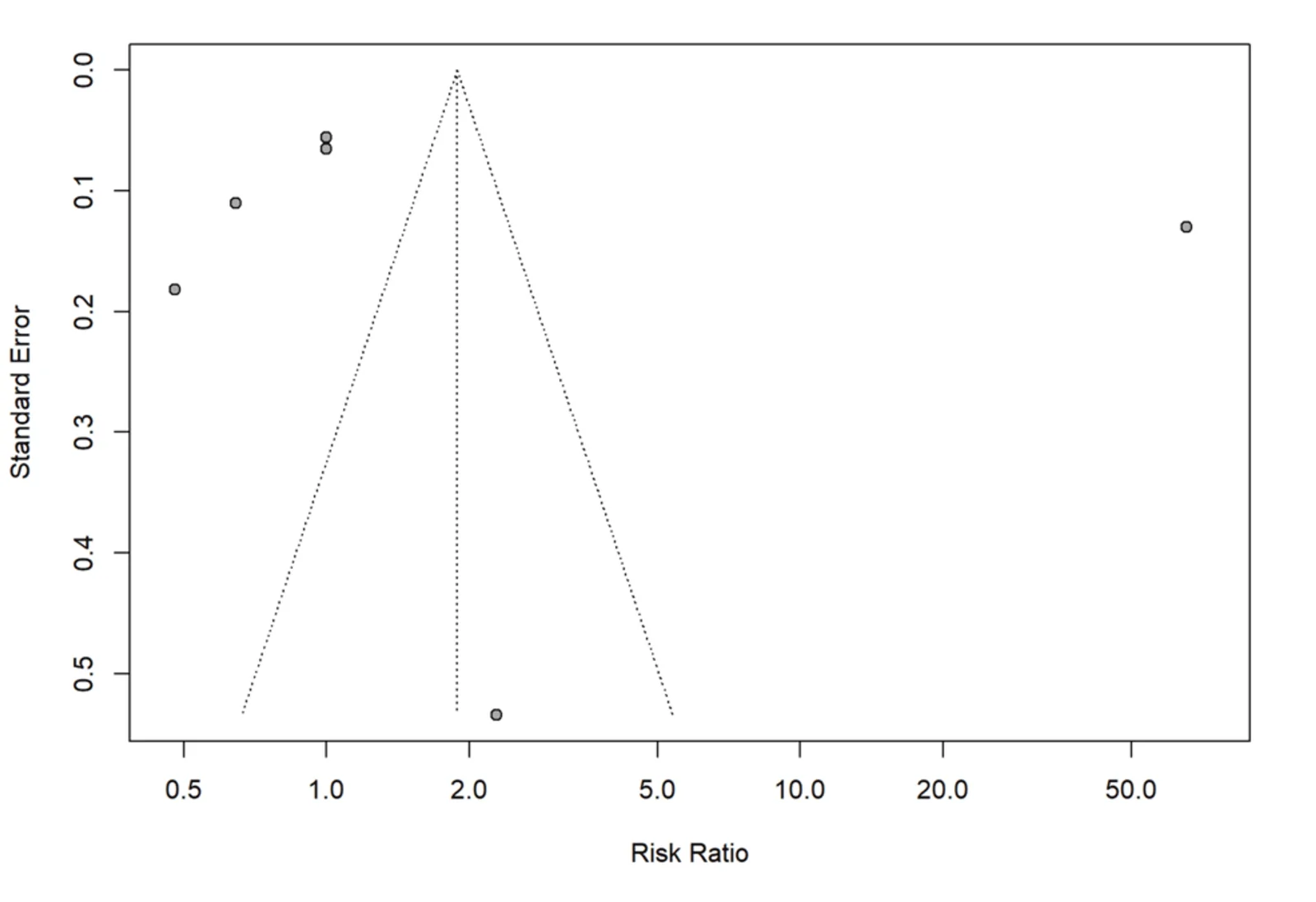
Funnel plot of publication bias of the included studies

Figure 4 shows a funnel plot for the assessment of publication bias and heterogeneity among the included studies. The studies are generally distributed on both sides of the pooled effect, with the more precise studies clustering toward the top of the plot, indicating stable effect estimates. A single study with a very large risk ratio appears on the far right and, together with a less precise study at the bottom, contributes to the apparent spread of the funnel. This pattern is more suggestive of between-study heterogeneity than true publication bias. Visual interpretation is limited because only a small number of studies were included. Consistent with the plot, Begg and Mazumdar’s rank correlation test was not statistically significant (z = -0.49, p = 0.6242), indicating no detectable small-study effects (Table 3).

**Table 3:** Begg’s test for statistical assessment of publication bias and heterogeneity

Method	Effect size	Standard Error	Test statistic	p-value	Conclusion
Rank correlation test (Begg & Mazumdar)	-2.0000	4.0825	z = -0.49	0.6242	Not significant; no detectable small-study effects

Patient-Reported Outcomes, Complications, and Clinical Outcomes.

**Figure 5:**
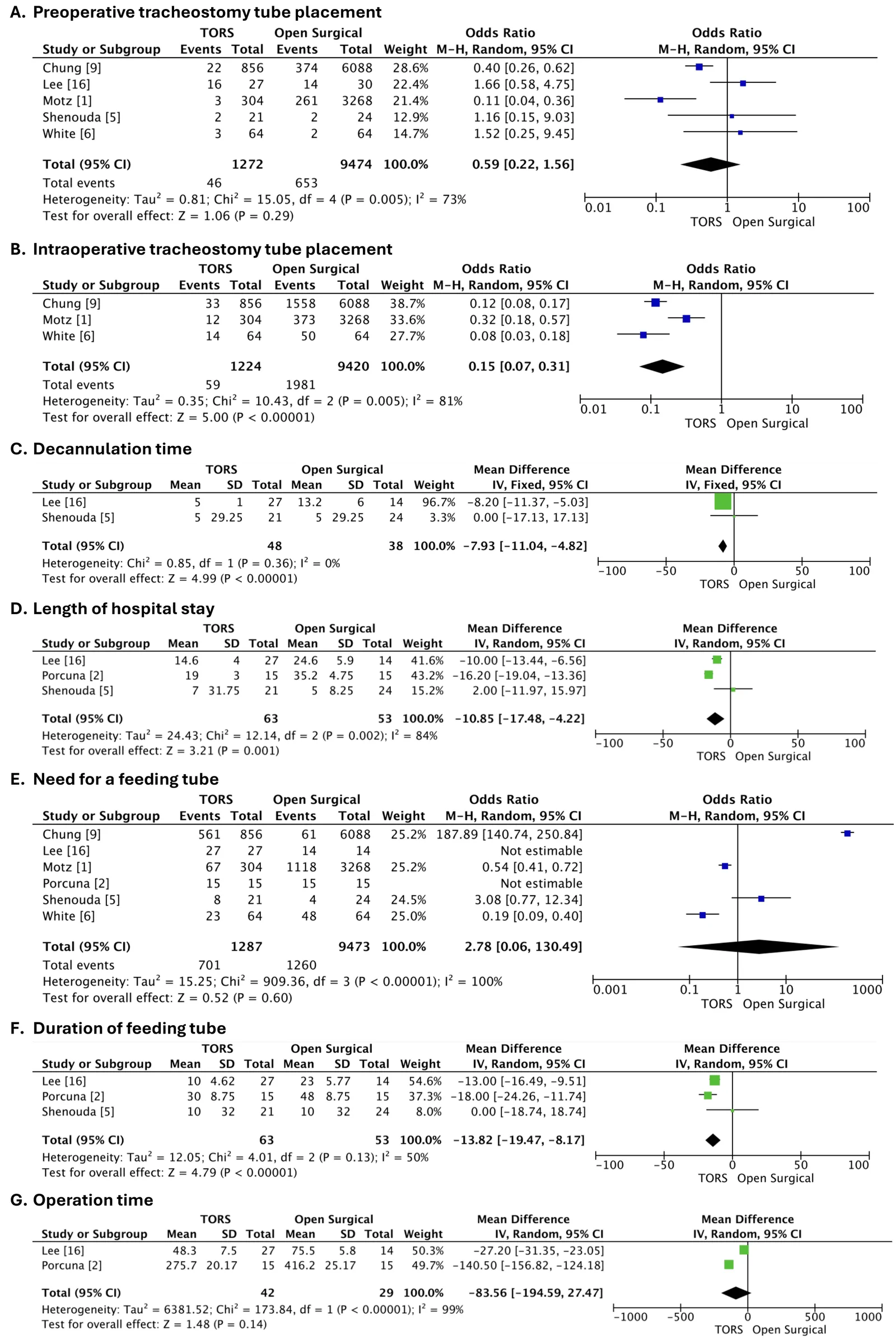
Forest plot for functional outcomes A. Preoperative tracheostomy tube placement, B. Intraoperative tracheostomy tube placement, C. Decannulation time, D. Length of hospital stay, E. Need for a feeding tube, F. Duration of the feeding tube, G. Operation time Sources: [1,2,5,6,9,16]

Functional outcomes: Five studies evaluated perioperative tracheostomy tube placement, yielding an odds ratio of 0.59 (95% CI: 0.22, 1.56; P = 0.29), indicating no statistically significant difference between TORS and open surgical approaches. In contrast, three studies assessing intraoperative tracheostomy tube placement showed a significant odds ratio of 0.15 (95% CI: 0.07, 0.31; P < 0.0001), demonstrating a clear advantage for TORS. Both analyses exhibited high heterogeneity (I² = 73% for perioperative and I² = 81% for intraoperative). Regarding decannulation time, two studies favored TORS with a mean difference of -7.93 (95% CI: -11.04, -4.82), and this finding was statistically significant (Z = 4.89, P < 0.0001). Three studies concerning the length of hospital stay (MD = -10.85, 95% CI -17.48, -4.22, P = 0.001) also favored TORS, though with high heterogeneity (I² = 84%). Regarding the need for a feeding tube, OR = 2.78, 95% CI: 0.06, 130.49; P = 0.60 suggested no significant difference but high heterogeneity (I² = 100%). However, the duration of feeding tube use showed a mean difference favoring TORS (MD= -13.82, 95% CI -19.47, -8.17, P < 0.00001), indicating a significant reduction in the length of time feeding tubes were needed for patients undergoing TORS compared to those undergoing open surgical approaches. Only two studies documented the operation time, and there was no difference between the two groups (MD = -83.56, 95% CI -194.59, 27.47, P=0.14) (Figure 5).

**Figure 6:**
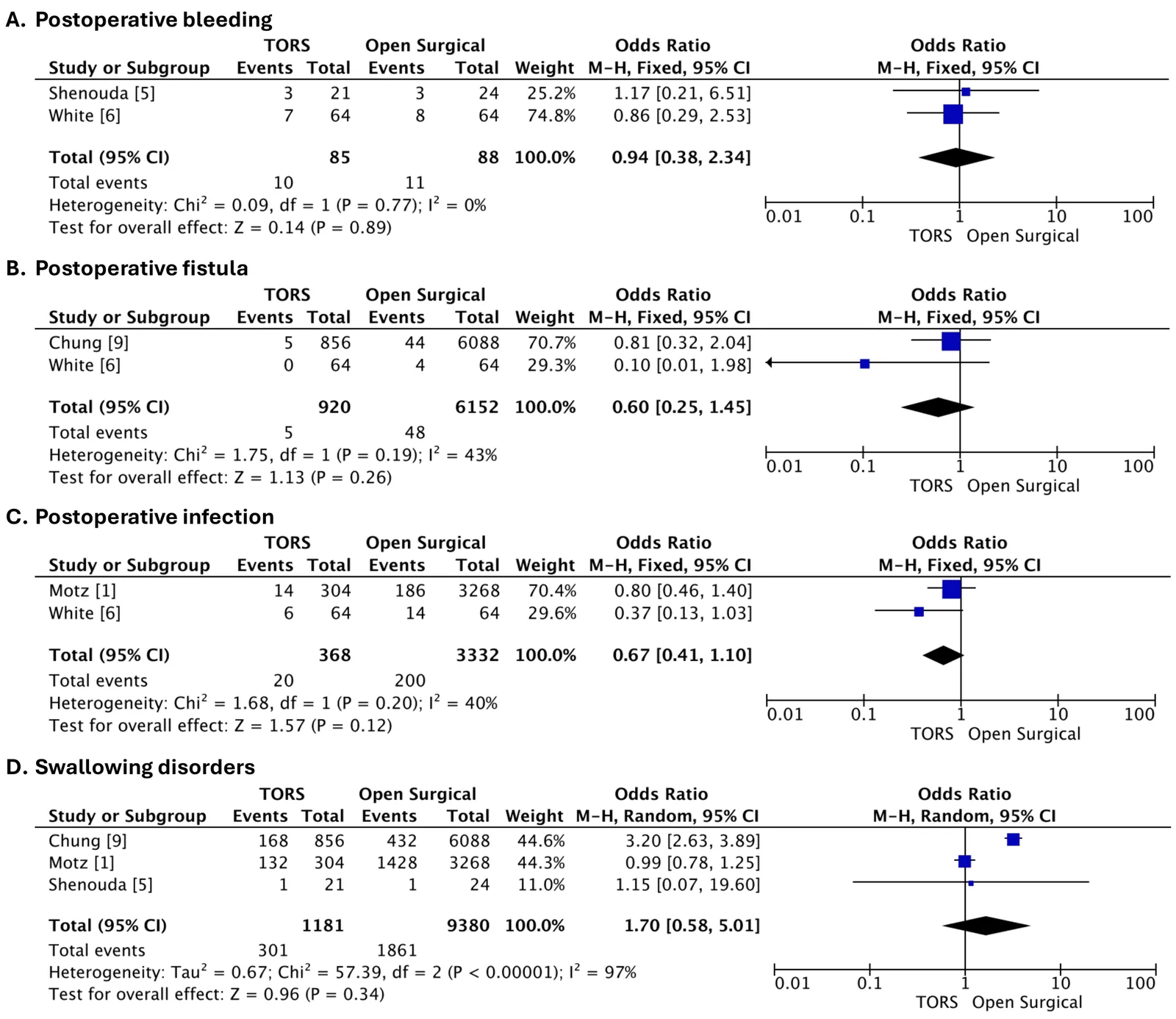
Forest plots of postoperative complications A. Postoperative bleeding, B. Postoperative fistula, C. Postoperative infection, D. Swallowing disorders Sources: [1,5,6,9]

Postoperative complications: Only two studies addressed postoperative complications, including bleeding (OR = 0.94, 95% CI 0.38, 2.34, P = 0.89), fistula formation (OR = 0.60, 95% CI 0.25, 1.45, P = 0.26), and infection (OR = 0.67, 95% CI 0.41-1.10, P = 0.12). None of these outcomes demonstrated statistically significant differences, and the analyses indicated low heterogeneity (I² < 50%). Three studies evaluated swallowing disorders (OR = 1.7, 95% CI 0.58, 5.01, P=0.34), which also showed no statistically significant differences between the two groups (Figure 6).

**Figure 7:**
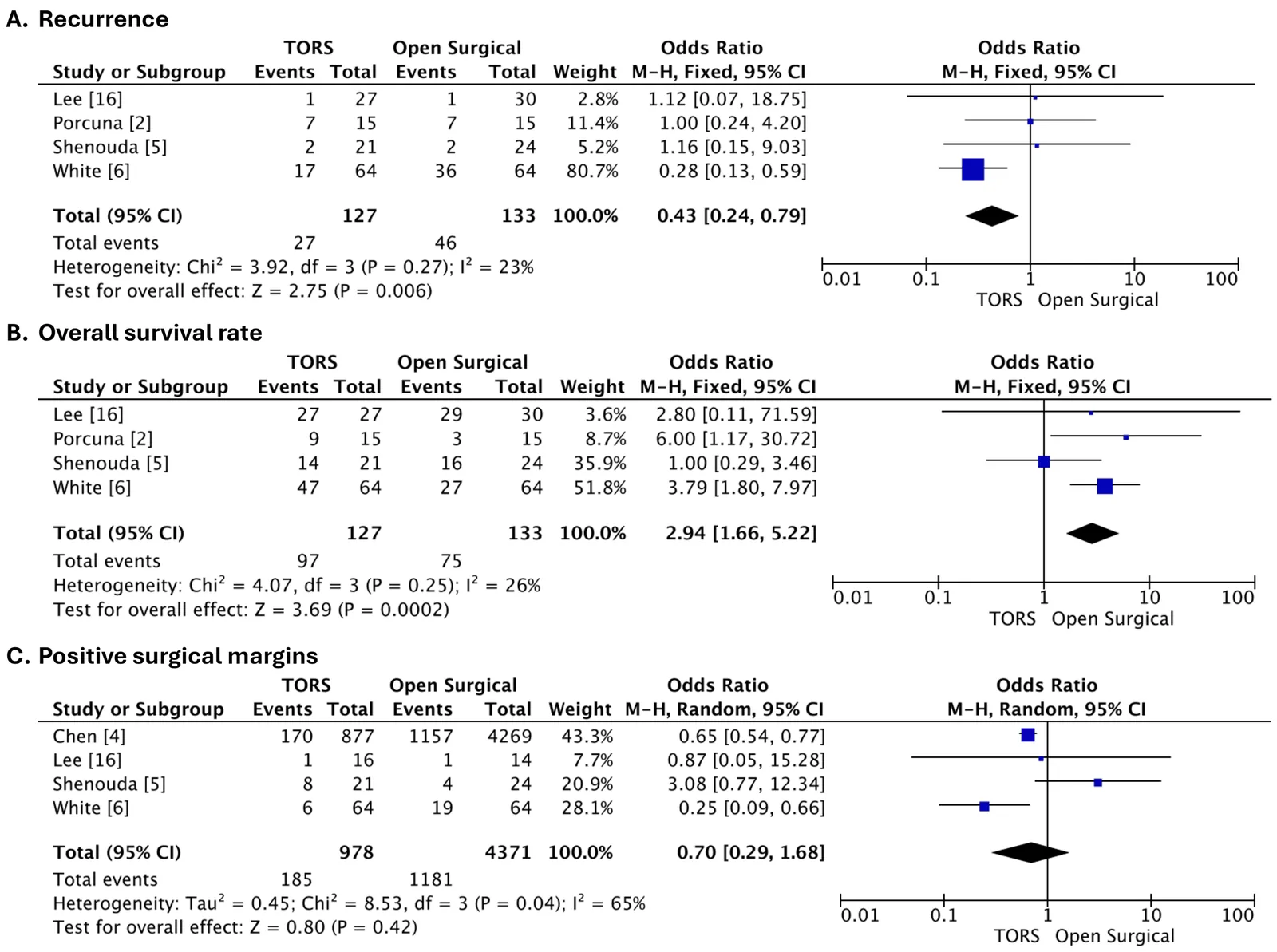
Forest plots of oncological outcomes A. Recurrence, B. Overall survival rate, C. Positive surgical margins Sources: [2,4-6,16]

Oncological outcomes: Recurrence rates were analyzed across four studies, revealing a significant difference between TORS and open surgical approaches. The odds ratio for recurrence was calculated at 0.43 (95% CI 0.24, 0.79, P = 0.006), indicating a lower likelihood of recurrence in the TORS group compared to open surgery. Additionally, overall survival rates showed a marked improvement with TORS, with an OR of 2.94 (95% CI 1.66, 5.22, P = 0.0002). Furthermore, there were four studies concerning the positive surgical margins; the odds ratio was 0.70 (95% CI 0.29, 1.68, P = 0.42), indicating no difference between the excision TORS and open surgical approach for OPSCC (Figure 7).

Discussion

This systematic review and meta-analysis evaluated the outcomes of TORS compared to open surgical approaches in patients ‎undergoing surgery for OPSCC. Our findings ‎highlight significant differences in functional and oncological outcomes. However, the two approaches had no significant differences in postoperative ‎complications or positive surgical margins.‎

Regarding the functional outcomes, our analysis revealed several significant ‎differences favoring TORS over open surgery. Specifically, TORS was associated with a ‎reduced need for a tracheostomy tube during surgery (odds ratio of 0.15) and a significantly ‎shorter decannulation time (mean difference of -7.93 days). These findings support the notion ‎that TORS may result in less postoperative airway management and more rapid respiratory function recovery, consistent with previous studies highlighting the ‎benefits of minimally invasive techniques in head and neck surgery [17]. These findings can be attributed to the transoral nature of TORS, which minimizes soft-tissue disruption and avoids the need for a mandibulotomy, thereby preserving normal anatomical structures, reducing postoperative edema, and facilitating earlier restoration of airway function.

The length of hospital stay was significantly shorter in the TORS group, further emphasizing the ‎efficiency and potential for faster recovery with this technique. This finding contrasts with previous systematic reviews and meta-analyses, which showed no significant ‎differences in the length of hospital stay between TORS and open surgery [7]. ‎This discrepancy may stem from the fact that the other review included TORS ‎and transoral laser microsurgery (TLM), which could have influenced the overall ‎results. Moreover, it is worth mentioning that TORS is associated with a lower need for free-flap reconstruction compared to open surgery. This finding is consistent with a previous ‎review, which also showed that TORS reduced the risk of free-flap reconstruction [8]. This ‎may be due to the enhanced visualization, magnification, and maneuverability provided by the robotic platform, which facilitate precise tumor resection while minimizing disruption of surrounding structures. However, the need for reconstruction is strongly influenced by tumor size, subsite, previous treatment, and the extent of resection. 

Despite the functional advantages of TORS, postoperative complications, including ‎bleeding, fistula formation, and infection, did not differ significantly between the two groups, consistent with a previous study reporting ‎similar rates of postoperative bleeding and fistula formation between TORS and open surgery [8]. Recent studies also suggest favorable postoperative outcomes following TORS, with a recent meta-analysis of HPV-associated OPSCC reporting pooled postoperative hemorrhage and tracheostomy rates of approximately 7% and 9%, respectively [18]. Interestingly, swallowing disorders also did not show a significant difference between ‎groups, indicating that TORS, despite its minimally invasive nature, may not provide a substantial advantage in preserving swallowing function. Although preservation of structures with TORS might theoretically improve swallowing function, postoperative swallowing is multifactorial and may be influenced by the extent and location of resection, reconstruction, baseline swallowing function, and the use of adjuvant radiotherapy or chemoradiotherapy. Previous research has demonstrated that swallowing outcomes after TORS can vary considerably according to treatment intensity and patient selection, emphasizing that the surgical approach represents only one determinant of long-term swallowing function [19].

From an oncological perspective, recurrence rates and overall survival demonstrated a clear benefit for ‎TORS, with a lower odds ratio for recurrence (OR = 0.43) and ‎higher overall survival (OR = 2.94). These findings are broadly consistent with previous studies reporting favorable oncological outcomes following TORS [17]. Another study has also reported favorable long-term survival after TORS, particularly in appropriately selected patients with HPV-associated OPSCC [20]. However, the observed survival advantage should be interpreted cautiously and may not be directly attributable to TORS itself. Patient selection is an important potential source of confounding, as patients undergoing TORS may have smaller, more accessible tumors, lower disease burden, better functional status, or other favorable prognostic characteristics. Furthermore, unmeasured confounding and selection bias may persist despite multivariable and propensity-score adjustment [17]. This is particularly relevant because more extensive tumors that cannot be adequately exposed or safely resected transorally are more likely to require an open surgical approach.

Importantly, positive surgical margins did not differ significantly between the two groups, ‎suggesting that TORS does not compromise the completeness of tumor resection. This finding is consistent with previous systematic reviews comparing TORS with open approaches [21].

This systematic review and meta-analysis have several limitations. First, the review ‎included a limited number of studies, and none were randomized controlled trials. Consequently, the findings may be influenced by selection bias and residual confounding. Second, some studies combined open surgical techniques with other non-‎robotic approaches, such as TLM and endoscopic procedures, ‎which could introduce variability in the results. Third, several important data points were ‎missing in the included studies, such as the histological type of cancer and HPV status, which ‎could have provided more comprehensive insights into the patient population. Lastly, some forest plots showed substantial heterogeneity in operative time, length of hospital stay, ‎and swallowing disorders. Differences in patient characteristics, tumor characteristics, and treatment approaches may have contributed to this heterogeneity. These variations may have impacted the overall conclusions. ‎Additional high-quality prospective clinical studies are needed to address these limitations and provide more robust evidence. ‎

## Conclusions

We have conducted a high-quality systematic review and meta-analysis comparing TORS with open surgery as an approach for OPSCC. Our findings suggest that TORS offers an advantage in functional outcomes, including reduced tracheostomy dependence, faster recovery, and shorter hospital stay. Although the recurrence rates were lower and the overall survival was higher in the TORS group, postoperative complications and positive surgical margins did not significantly differ between the two groups. However, substantial variation does exist across different studies in terms of patient selection, surgical technique, and follow-up duration. Further research, particularly well-designed prospective studies and randomized controlled trials with longer follow-up periods, is necessary to refine patient selection criteria and to optimize the surgical approaches and strategies. Appropriate case selection and the surgeon's expertise will remain critical for achieving optimal outcomes.

## References

[1] Motz K, Chang HY, Quon H, Richmon J, Eisele DW, Gourin CG (2017). Association of transoral robotic surgery with short-term and long-term outcomes and costs of care in oropharyngeal cancer surgery. JAMA Otolaryngol Head Neck Surg.

[2] Virós Porcuna D, Viña Soria C, Vila Poyatos J (2023). Oropharyngeal free flap reconstruction: transoral robotic surgery versus open approach. Laryngoscope Investig Otolaryngol.

[3] Chillakuru Y, Benito DA, Strum D (2021). Transoral robotic surgery versus nonrobotic resection of oropharyngeal squamous cell carcinoma. Head Neck.

[4] Chen MM, Roman SA, Kraus DH, Sosa JA, Judson BL (2014). Transoral robotic surgery: a population-level analysis. Otolaryngol Head Neck Surg.

[5] Shenouda K, Rubin F, Garcia D, Badoual C, Bonfils P, Laccourreye O (2020). Evaluation of robotic surgery for transoral resection of T1-2 squamous cell carcinoma of the tonsillar fossa. Eur Ann Otorhinolaryngol Head Neck Dis.

[6] White H, Ford S, Bush B (2013). Salvage surgery for recurrent cancers of the oropharynx: comparing TORS with standard open surgical approaches. JAMA Otolaryngol Head Neck Surg.

[7] Chen H, Liu Y, Huang D, Zhang X, She L (2023). Transoral robotic surgery vs. non-robotic surgeries for oropharyngeal squamous cell carcinoma: systematic review and meta-analysis. J Robot Surg.

[8] Park DA, Lee MJ, Kim SH, Lee SH (2020). Comparative safety and effectiveness of transoral robotic surgery versus open surgery for oropharyngeal cancer: a systematic review and meta-analysis. Eur J Surg Oncol.

[9] Chung TK, Rosenthal EL, Magnuson JS, Carroll WR (2015). Transoral robotic surgery for oropharyngeal and tongue cancer in the United States. Laryngoscope.

[10] (2025). PROSPERO: CRD42024616159. Comparative outcomes of transoral robotic surgery versus open surgery in head and neck cancer: a systematic review and meta-analysis. https://www.crd.york.ac.uk/PROSPERO/view/616159.

[11] Page MJ, McKenzie JE, Bossuyt PM (2021). The PRISMA 2020 statement: an updated guideline for reporting systematic reviews. BMJ.

[12] Thompson M, Tiwari A, Fu R, Moe E, Buckley DI (2012). A Framework To Facilitate the Use of Systematic Reviews and Meta-Analyses in the Design of Primary Research Studies. https://pubmed.ncbi.nlm.nih.gov/22299187/.

[13] Ouzzani M, Hammady H, Fedorowicz Z, Elmagarmid A (2016). Rayyan--a web and mobile app for systematic reviews. Syst Rev.

[14] Slim K, Nini E, Forestier D, Kwiatkowski F, Panis Y, Chipponi J (2003). Methodological index for non-randomized studies (minors): development and validation of a new instrument. ANZ J Surg.

[15] Downes MJ, Brennan ML, Williams HC, Dean RS (2016). Development of a critical appraisal tool to assess the quality of cross-sectional studies (AXIS). BMJ Open.

[16] Lee SY, Park YM, Byeon HK, Choi EC, Kim SH (2014). Comparison of oncologic and functional outcomes after transoral robotic lateral oropharyngectomy versus conventional surgery for T1 to T3 tonsillar cancer. Head Neck.

[17] Nguyen AT, Luu M, Mallen-St Clair J (2020). Comparison of survival after transoral robotic surgery vs nonrobotic surgery in patients with early-stage oropharyngeal squamous cell carcinoma. JAMA Oncol.

[18] Hajihosseni H, Hajitaghizadeh R, Sharifi HR, Lin D (2026). Oncologic, functional, and economic outcomes of transoral robotic surgery for HPV-associated oropharyngeal squamous cell carcinoma: a systematic review and meta-analysis of pathology and treatment strategies. J Robot Surg.

[19] Hughes RT, Levine BJ, May N (2023). Survival and swallowing function after primary radiotherapy versus transoral robotic surgery for human papillomavirus-associated oropharyngeal squamous cell carcinoma. ORL J Otorhinolaryngol Relat Spec.

[20] Yver CM, Shimunov D, Weinstein GS (2021). Oncologic and survival outcomes for resectable locally-advanced HPV-related oropharyngeal cancer treated with transoral robotic surgery. Oral Oncol.

[21] Roselló À, Albuquerque R, Roselló-Llabrés X, Marí-Roig A, Estrugo-Devesa A, López-López J (2020). Transoral robotic surgery vs open surgery in head and neck cancer. A systematic review of the literature. Med Oral Patol Oral Cir Bucal.

